# Protein/DNA interactions in complex DNA topologies: expect the unexpected

**DOI:** 10.1007/s12551-016-0241-7

**Published:** 2016-11-14

**Authors:** Agnes Noy, Thana Sutthibutpong, Sarah A. Harris

**Affiliations:** 1 Department of Physics, Biological Physical Sciences Institute, University of York, York, YO10 5DD UK; 2 Theoretical and Computational Physics Group, Department of Physics, King Mongkut University of Technology Thonburi, 126 Pracha Uthit Road, Bang Mod, Thung Khru, Bangkok, Thailand 10140; 3 School of Physics and Astronomy, University of Leeds, 192 Woodhouse Lane, Leeds, UK LS2 9JT; 4 Astbury Centre for Structural and Molecular Biology, University of Leeds, 192 Woodhouse Lane, Leeds, UK LS2 9JT

**Keywords:** DNA supercoiling, DNA topology, DNA-binding proteins, Computer simulations, Thermodynamics, DNA structural organisation

## Abstract

DNA supercoiling results in compacted DNA structures that can bring distal sites into close proximity. It also changes the local structure of the DNA, which can in turn influence the way it is recognised by drugs, other nucleic acids and proteins. Here, we discuss how DNA supercoiling and the formation of complex DNA topologies can affect the thermodynamics of DNA recognition. We then speculate on the implications for transcriptional control and the three-dimensional organisation of the genetic material, using examples from our own simulations and from the literature. We introduce and discuss the concept of coupling between the multiple length-scales associated with hierarchical nuclear structural organisation through DNA supercoiling and topology.

## Introduction

We propose that supercoiling provides a mechanism to amplify information and to communicate activity in the genome across multiple levels of nuclear organisation. In support of this view, we combine the physical insight provided by computer simulations that show in atomic detail how DNA responds to topological stress with experimental evidence for the importance of supercoiling. Supercoiling effects DNA interactions with other molecules across a spectrum of length-scales, from the size of the counterion species in the environment to up to nuclear domains formed by clusters of DNA-binding proteins. We conclude that an understanding of the physical interactions that couple chromosome organisation to gene regulation will require a multi-scale approach starting from sequence-dependent DNA mechanics, through the organisation of DNA domains by architectural DNA-binding proteins up to the global organisation of the whole chromosome.

In this review, we first describe the origins of supercoiling in DNA, and then the multiple levels of hierarchical DNA structural organisation from a DNA mechanics point of view. The effect of the counterion environment and DNA-binding proteins on supercoiling locally and globally are then discussed in the context of each level of nuclear organisation. The background concepts in DNA supercoiling and topology, such as definitions of twist and writhe and superhelical density, can be found in Bates and Maxwell (Bates and Maxwell 2005), and a detailed description of the evidence for the importance of DNA supercoiling in mediating protein–DNA interactions can be found in a previous review (Fogg et al. 2012).

## Origins of supercoiling in the genome

DNA supercoiling is a cellular strategy for packing the genetic material efficiently into a small nuclear space, but it is also implicated in genetic control (see, for examples, Fogg et al. 2012; Gilbert and Allan 2014; Koster et al. 2010; Lavelle 2014). The physical mechanisms that couple supercoiling to levels of gene transcription are less well understood than the biochemical signalling pathways mediated by DNA-binding proteins such as transcription factors, activators and repressors, because of the experimental difficulties associated with measuring supercoiling in active DNA. The origins of DNA supercoiling can be considered to consist of static supercoils, which change slowly and are externally regulated by the cell, and dynamic supercoils, which are introduced transiently by DNA processing machines, such as RNA polymerase.

### Static DNA supercooling

In prokaryotes, the genome is maintained in a negatively supercoiled state by DNA gyrase, which uses chemical energy to maintain an average torsional stress of around σ = −0.06 within the DNA (Collin et al. 2011). In eukaryotes, the left-handed wrapping of the DNA in the nucleosome constrains the DNA to be negatively writhed; this writhe is converted into supercoiling if the histone complex is displaced (Teves and Henikoff 2014). Although the higher order organisation of chromatin remains poorly understood, levels of superhelical density in the range σ = −0.09 and −0.06 range have been suggested for chromatin fibres, depending upon the precise organisation of the nucleosome units (Norouzi and Zhurkin 2015). Magnetic tweezer experiments that subjected nucleosome arrays to torque have demonstrated that chromatin can absorb a large amount of supercoiling without undergoing a substantial change in length. This ability of chromatin to act as a “topological buffer” that shields regions of the genome from changes in supercoiling could be explained by a model in which chromatin adopts multiple conformational states (Bancaud et al. 2006).

### Dynamic DNA supercoiling

Dynamic supercoiling is introduced by transcription, as separation of the double-helical strands creates positive and negative supercoiling ahead and behind the polymerase complex, respectively (Liu and Wang 1987). If the ends of the DNA are restrained, then this dynamic supercoiling will be stored within this section of the genome. Transcription is stalled whenever too much positive supercoiling builds up ahead of the RNA polymerase and its associated machinery while a gene is being read (Chong et al. 2014). While the presence of topoisomerases (which relax supercoiled DNA) implies that the cell has mechanisms in place to dissipate dynamic supercoiling (Baranello et al. 2016), experiments that measured the levels of negative supercoiling in the DNA using intercalating agents detected superhelical stresses from transcription equivalent to σ = −0.07 over length scales of between 1 and 1.5 kb from the polymerase enzyme (Kouzine et al. 2013), as well as a large negative supercoiling gradient between the replication origin and the terminus during stationary growth phase in *Escherichia coli* (Lal et al. 2016).

There is growing evidence that dynamic supercoiling confers regulatory information over long distances through the genome. In general, genes that are AT rich tend to be downregulated by increased negative supercoiling, whereas GC-rich genes have a propensity to be upregulated (for more details see the review by Fogg et al. 2012). Negative supercoiling destabilises the double-helical structure of the DNA, which facilitates melting and therefore affects the delicate balance between DNA opening and reannealing required for successful transcription. This provides a mechanism to couple together the transcriptional activity of successive genes without the requirement for DNA-binding proteins. Transmission of information through the DNA itself provides a particularly efficient mechanism for co-operative gene expression, as it does not rely either on protein production or the location of a specific binding site by protein diffusion. A striking demonstration of the importance of coupled gene expression comes from an analysis of the *E. coli* genome, leading to the suggestion that genes are positioned and orientated to ensure that the influence of supercoiling from transcription of neighbouring genes is maintained through evolution (Sobetzko 2016). Studies in eukaryotes have additionally suggested that the generation of short divergent RNA transcripts could have a regulatory function by underwinding the promoters and facilitating transcription (Naughton et al. 2013a).

The amount of superhelical stress that builds up in the DNA from transcription, the length-scale associated with this mechanical perturbation and its timescale depends upon a complex interplay between a number of physical effects: the efficiency of supercoil removal by topoisomerases, the mechanical response of the DNA itself and the presence of DNA-binding proteins which may act as mechanical clamps imposing a given global topology. Static and dynamic supercoiling can also be coupled. For example, negative supercoiling can be generated by unwinding DNA from histones, which can then facilitate promotor melting and passage of RNA polymerase along the DNA (Kouzine et al. 2014). The connection between DNA–protein interactions and static and dynamic supercoiling introduces a coupling between the multiple levels of structural organisation within the genome, which is discussed in the following section.

## A mechanical view of hierarchical DNA structural organisation

Just as primary protein sequences are folded into secondary and tertiary structures, which at the quaternary level of structural organisation are then arranged within larger protein complexes, the nuclear material in both prokaryotes and eukaryotes is packaged into the nuclear region in a hierarchical manner. The close coupling between the various organisational regimes makes any classification somewhat subjective; in the view we present here (see Table 1) the emphasis is on the role of DNA mechanics in determining the overall three-dimensional (3D) structure of genomes. A structural description of the hierarchy of eukaryotic chromatin is provided by a recent review (Ozer et al. 2015).

**Table 1:** The hierarchical levels of DNA structural organisation in prokaryotic and eukaryotic genomes

Level A:	
DNA sequence (e.g. Travers et al. 2012) and epigenetics (e.g. Breiling and Lyko 2015)	
Level B:	
Supercoiled DNA (plectonemes, toroids, melted regions) (e.g. Lavelle 2014)	
Prokaryotes	Eukaryotes
Level C: DNA architectural proteins (HU/FIS) (e.g. Travers and Muskhelishvili 2007)	Level C1: Nucleosome structure (e.g. Wu et al. 2010)
	Level C2: Polymorphic structure of 30-nm fibre (e.g. Norouzi and Zhurkin 2015)
Level D: Supercoiling domains (e.g. Le et al. 2013)	Level D: Supercoiling domains (e.g. Naughton et al. 2013b)
Level E: Topological domains (e.g. Badrinarayanan et al. 2015)	Level E: Topologically associated domains (e.g. Gilbert and Allan 2014)

*FIS* Factor of inversion stimulation,* HU* heat-unstable nucleoid protein

### DNA sequence

The primary structural organisation level of nuclear structure (level A in Table 1) is the DNA sequence itself, including epigenetic modifications in eukaryotes. The shape and flexibility of DNA is not uniform, as demonstrated by a series of X-ray crystallographic data (Hays et al. 2005; Olson et al. 1998). Rather, specific DNA sequences are known to be associated with particular shapes (Peters and Maher 2010), such as the curvature of DNA A-tracts (Haran and Mohanty 2009), and these shapes can introduce regions of high bending flexibility, such as TA steps (Haran and Mohanty 2009; Johnson et al. 2013; Tolstorukov et al. 2007). DNA chemical modifications which change the local DNA mechanics have also been suggested as basic regulatory mechanisms of gene expression. For example, epigenetic modifications such as methylation at CpG steps may increase DNA stiffness (Pérez et al. 2012) and impair nucleosome wrapping (Portella et al. 2013), suggesting that this mechanical signal may regulate promoters contained in so-called “CpG islands”.

### Plectonemic DNA

**Fig. 1:**
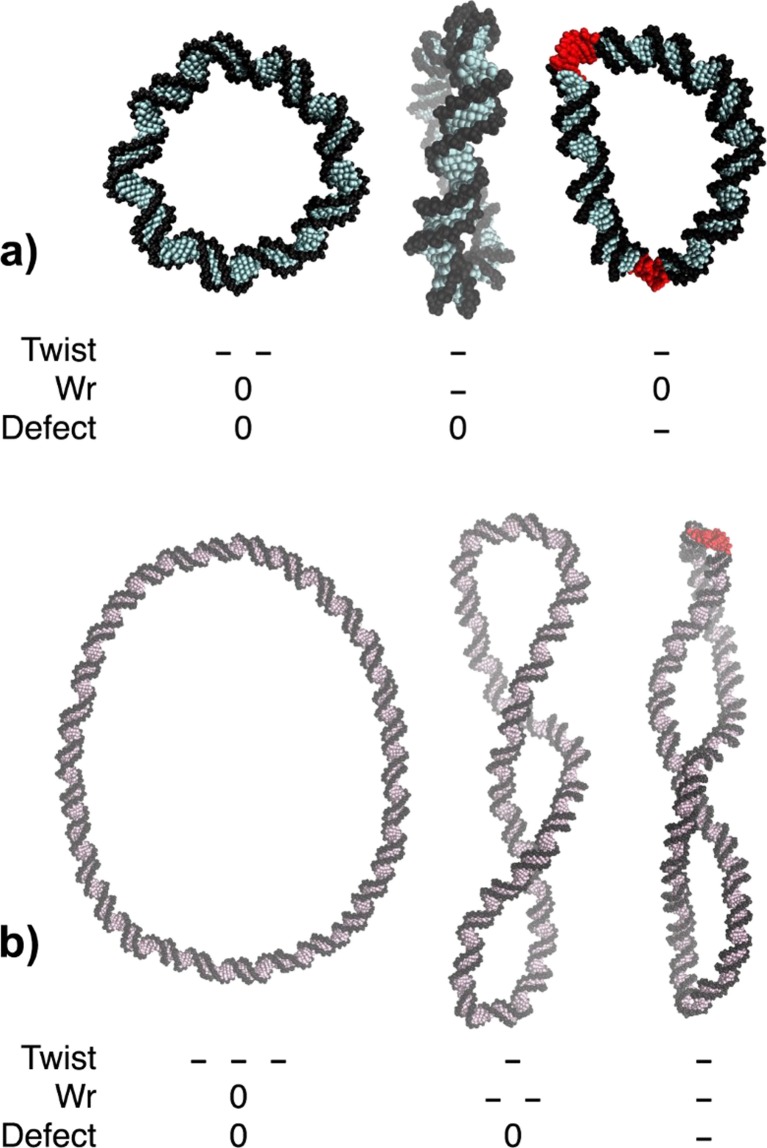
**a** Representative structures from an atomistic molecular dynamics (MD) simulation of a 108-bp negatively supercoiled DNA minicircle (σ ≈ −0.1): *left* untwisted planar circular conformation, *middle* negatively writhed (*Wr*) conformation, *right* relief of superhelical stress by defect formation (*Defect*; in *red*). **b** Representative structures from an atomistic MD simulation of a 336-bp negatively supercoiled DNA minicircle (σ ≈ −0.1): *left* untwisted planar circular conformation, *middle* negatively writhed (*Wr*) conformation, *right* partial superhelical stress relaxation due to defect formation (*Defect*; in *red*).* Minus signs* indicate the degree of untwisting or writhing (e.g.* Twist - - -* indicates that the circle is underwound by three turns)

Prokaryotic and eukaryotic DNA is generally stored under negative torsional stress. At the next level up in the structural hierarchy (level B in Table 1), the DNA adopts a plectonemic 3D conformation that is dependent upon the mechanics of its underlying sequence (Travers et al. 2012; van Loenhout et al. 2012), the levels of superhelical stress (Lavelle 2014; Salerno et al. 2012) and the positions of the topological restraints that maintain this superhelical tension (Czapla et al. 2013; Wei et al. 2014). The salt environment and the presence of kinks, denatured regions of DNA and higher-order structures such as cruciforms or quadruplexes, which are all favoured by the presence of supercoiling, will also contribute to the 3D conformation. Cruciform extrusion and quadruplex formation alters the DNA mechanics, releases superhelical stress and presents a dramatically different potential binding target for proteins and small molecules (see Fogg et al. 2012 and references therein). Atomistic molecular dynamics (MD) simulations of 108- and 336- bp DNA minicircles have detected structural disruptions in DNA at the apices of plectonemic supercoils which allow the DNA to partially unwrithe back towards a planar conformation [see Fig. 1a (Sutthibutpong et al. 2016) and Fig. 1b (Irobalieva et al. 2015)].

### Structural DNA–protein complexes

The presence of regulatory DNA-binding proteins, structural nuclear-associated proteins such as histones (in eukaryotes) or HU or FIS (in prokaryotes), topology and chromatin-processing DNA motors (level C in Table 1) and structural protein complexes which anchor the nuclear material to a fixed location within the cell (levels D and E in Table 1) are central to the structural organisation of all genomes (Gilbert and Allan 2014). However, there is considerable uncertainty as to the spatial organisation of DNA/protein units containing many nucleosomes (in eukaryotes) or arrays of HU/FIS complexes (in prokaryotes). From a mechanical viewpoint, the preferred conformations will be a result of the interplay between the structure and flexibility of the naked supercoiled DNA, and the identity of the structural proteins involved.

### External attachment of the nuclear material

At the largest organisational length-scale, attachment to fixed locations external to the nuclear material is governed by global cellular control mechanisms that are coupled to the cell cycle (Bickmore and van Steensel 2013). Microscopy studies using fluorescent in situ hybridisation, combined with experimental data based on chromosome capture technology (known as 3C, 4C or Hi-C), have shown that the genome is folded into discrete globular territories (Dekker et al. 2013; Nora et al. 2012; Williamson et al. 2014), but that these territories are highly dynamic and being continually remodelled (Nagano et al. 2013). Interpretation of these Hi-C contact maps in conjunction with measurements of the range of dynamic supercoiling from transcription (Dixon et al. 2012; Naughton et al. 2013b) have suggested that the genome is compartmentalised into smaller supercoiling domains of between 1.5 and 100 kb (see level D in Table 1) that exist within larger 1-Mb loops, known as topologically associated domains (TADS) (level E in Table 1) (Gilbert and Allan 2014). This achieves a particularly intimate coupling between the global and local organisation of the DNA and its associated proteins because the positions of topological restraints in turn control the distribution and maintenance of static and dynamic supercoiling (Badrinarayanan et al. 2015; Sobetzko 2016).

## DNA counterions and topology

### Effect of counterions at the base pair level

The salt environment of the DNA arguably involves the smallest length-scale that determines DNA topology (an individual Na^+^ cation has a van der Waals of around 1 Å). That electrostatic energy is such a crucial factor in determining the topology of the molecule of DNA is due to its polyelectrolyte nature. The concentration and chemical identity of the DNA counterion environment can significantly affect the equilibrium DNA twist, which implies that it is a key determinant of the superhelical density within any closed DNA topology (Fogg et al. 2006; Xu and Bremer 1997). In general, increasing the cation concentration results in more highly twisted DNA because the backbone phosphate charges along the DNA strand are screened more effectively. For example, Na^+^ and K^+^ ions overtwist the DNA by 0.03°/bp and 0.11°/bp at 40 mM, respectively. NH_4_^+^ has the strongest effect amongst the monovalent ions (0.19°/bp). Divalent ions overtwist DNA more than monovalent ions at low salt concentrations (0.44°/bp for 40 mM Ca^2+^). At higher salt concentrations (>50 mM), divalent ions also promote the crossing between DNA segments due to an excess positive charge at each divalent ion binding site (Xu and Bremer 1997).

The atomistic arrangement of counterions around supercoiled DNA cannot be observed directly by experimental means. Microsecond atomistic MD calculations of relaxed 18-bp linear DNA sequences interacting with K^+^ ions in water have shown that individual counterions ions bind at discrete sequence-dependent positions within the major and minor grooves, implying firstly that counterion condensation is intimately connected with DNA structure at atomistic and base pair levels, but also that repetitive DNA sequences can be templates for the formation of regular arrays of counterions (Pasi et al. 2015). Condensation of DNA (Teif and Bohinc 2011) has also been shown to require specific DNA–ion bridging interactions, which may be highly dependent on the helical repeat as the recognition between two DNA segments with similar helical twists and/or groove sizes should be preferable (Kornyshev and Leikin 2013). This has been hypothesised to drive the recombination of homologous DNA, which is crucial for gene shuffling and DNA repair (Lee et al. 2015).

### Effects of counterions on knotted and plectonemic DNA

X-ray crystal structures and MD simulations have shown that counterion–DNA interactions influence the global shape of DNA, in many cases more significantly and with greater specificity than would be expected from simple charge screening. DNA knotting experiments have demonstrated that specific counterion identities affect knotting probability by controlling the effective DNA helical diameter. The effective helical diameters of DNA segments in a divalent counterion environment can be reduced to 2 nm at high salt concentrations, which almost brings the two DNA segments into direct contact. For monovalent ions, 10- to 50-fold higher salt concentrations are needed to reproduce the equivalent experimental conditions (Shaw and Wang 1993). There is even an indirect dependence on the chemical nature of the anion through its affinity for its oppositely charged partner (Savelyev and Papoian 2007). In eukaryotes, chromatin structure has also been observed to be dependent on the salt environment using sedimentation velocity measurements and computational models. The most compact fibers appeared in the presence of multivalent cations such as Mg^2+^ or Co(NH_3_)_6_^3+^ due to the particularly effective charge screening between nucleosomal and linker DNA provided by these ion species (Korolev et al. 2010).

In plectonemic DNA, the electrostatic repulsion incurred at the crossing points and at the highly bent apices of the plectoneme ends is reduced through screening by positively charged ions. Consequently, switching the counterion environment can change the twist/writhe partition in supercoiled DNA. Electron microscopy, atomic force microscopy (AFM) (Bednar et al. 1994; Bussiek et al. 2003; Cherny and Jovin 2001; Shlyakhtenko et al. 2003) and computer modelling at the both the polymer (Giovan et al. 2014; Schlick et al. 1994; Zheng and Vologodskii 2009) and atomistic level (but within a continuum solvent approximation) (Mitchell and Harris 2013) has shown that positively charged counterions promote the compaction of supercoiled DNA into complex topologies in supercoiled plasmids.

**Fig. 2:**
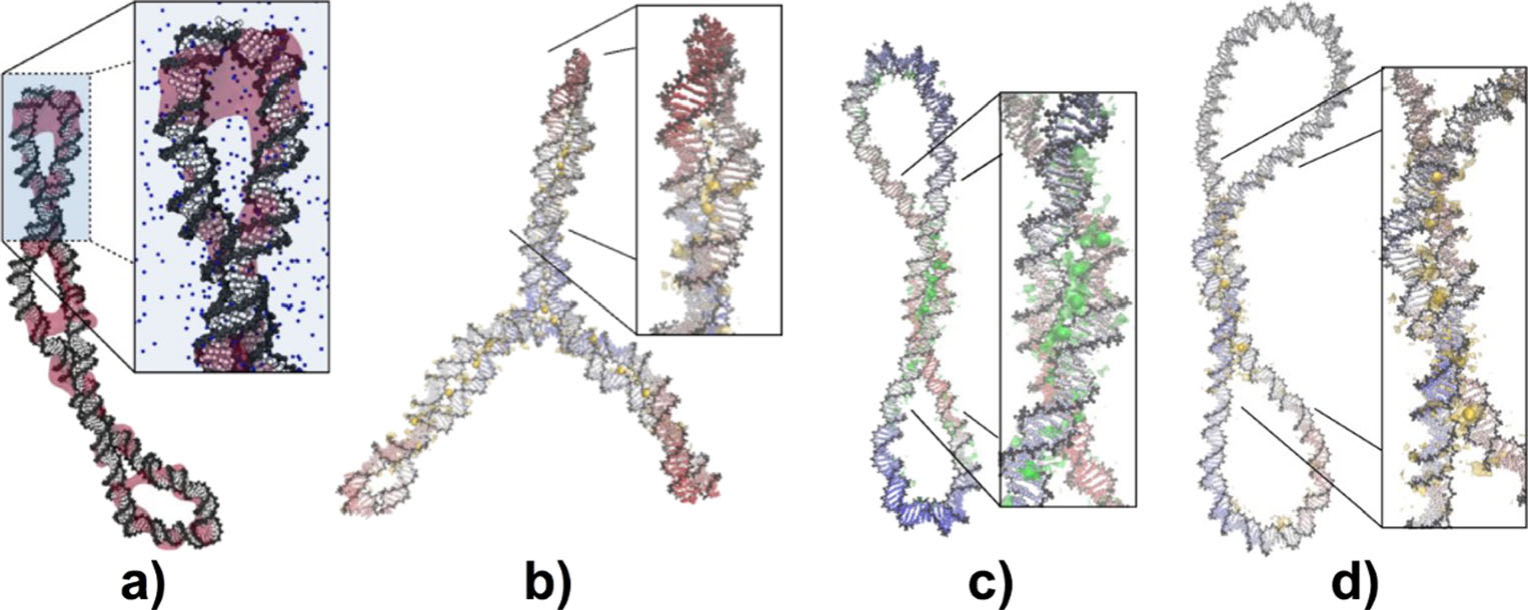
Regions of high positive counterion density around negatively supercoiled DNA minicircles. **a** Structure from explicitly solvated atomistic MD of a negatively supercoiled 336-bp minicircle (σ ≈ −0.1) showing regions highly populated by counterions over 20 ns as *pink* isosurfaces. **b**–**d** Averaged structure of a 339-bp minicircle solvated in 100 mM Ca(Cl)_2_ (highly negatively supercoiled; σ ≈ −0.2) (**b**), of a 260-bp minicircle in 200 mM NaCl (σ ≈ −0.08) (**c**) and of a 339-bp minicircle in 100 mM Ca(Cl)_2_ (σ ≈ −0.07) (**d**) obtained from a superposition of 1000 snapshots corresponding to the last 10 ns of 100-ns MD trajectories. Ca^2+^ density peaks are shown in *yellow* and Na^+^ peaks in *green*

Insight into the physical mechanisms that determine the salt dependence of the twist/writhe partition, the importance of the sequence-dependent DNA twist (for a detailed discussion see Bates et al. 2013 and Sutthibutpong et al. 2015) and distributions of individual cations has been provided by MD simulations of DNA minicircles in an explicit counterion and water environment. In trajectories of minicircles over a range of constantly maintained superhelical densities (from values as high as σ = −0.2 to those around σ = −0.07, which are closer to in vivo conditions; Zechiedrich et al. 2000), both monovalent Na^+^ and divalent Ca^2+^ cations cause condensation of the DNA to such an extent that at the crossing points individual counterions are simultaneously bound within the grooves of adjacent DNA strands, as shown in Fig. 2. These simulations have also detected increased counterion density at the tightly bent apices of these tiny DNA loops. Figure 2c shows a kink formed at the plectoneme tips of 336- and 339-bp negatively supercoiled DNA minicircles, where the counterions are crowded underneath the kinked structure, and individual counterions have been observed to be bound at a DNA kink site (Mitchell et al. 2011). The presence of the mobile counterion atmosphere promotes DNA kinking because it increases in density at the strands get closer and offsets the free energy required to break the double helical structure. Conversely, in simulations of supercoiled DNA in which bending and writhing were not permitted during the calculations, DNA regions in which bases flipped out of the double helix were associated with a reduction in counterion density due to the lower electrostatic potential at the defect (Randall et al. 2009).

Another striking example of how local counterion-mediated DNA interactions can have a global effect is provided by a structural analysis of DNA structures containing crossing points in the archive of the Protein Data Bank (PDB). Left-handed crossovers, which are associated with negatively supercoiled DNA, were found to be inherently less stable than their right-handed counterparts because in the latter the backbone of one strand can fit into the major groove of the other in the presence of bridging Mg^2+^ counterions (Várnai and Timsit 2010). This result shows that specific counterion–DNA interactions within the grooves can increase the asymmetry between the energetics of positively and negatively supercoiled DNA plectonemes.

### Counterions exert multi-scale effects through DNA topology

The overview provided by these experiments and simulations shows that the interaction between DNA and its counterion environment influences multiple length-scales in the DNA organisational structural hierarchy. Counterions bind to the grooves of DNA in a specific manner that depends on charge density, cation size and the width of the DNA major and minor grooves, which in turn depends on both supercoiling and on the DNA sequence. This counterion density then controls the partitioning between twist and writhe within closed DNA topologies and, consequently, the global shape of that section of DNA. Writhed structures and kinks are stabilised by counterion screening which can be sufficiently strong that neighbouring DNA strands are effectively bound together by a counterion bridge that self-assembles within the grooves. The coupling between different length-scales introduced by counterion condensation is increased by the restraint of the DNA polymer into a closed topology. For example, in unrestrained linear sequences, the DNA can simply rotate in response to salt-induced changes in twist, whereas in a closed topology this will affect the global superhelical stress. While the compactness of long linear DNA sequences would also be expected to increase with increased electrostatic screening, in supercoiled DNA these changes can be dramatic because the increased superhelical stress can introduce kinks and defects which massively increase the local flexibility of the DNA. Therefore, the imposition of a closed DNA topology amplifies the effect of any changes in counterion condensation from the level of the DNA grooves up to the global shape of a given plectonemic region. There is also the potential for the long-range interaction between distal sites to be sequence specific, which adds another level of coupling between individual base pairs and global shape within a given topologically closed region.

## Specific and non-specific DNA–protein interactions in complex topologies

### Proteins as “complex cations”

**Fig. 3:**
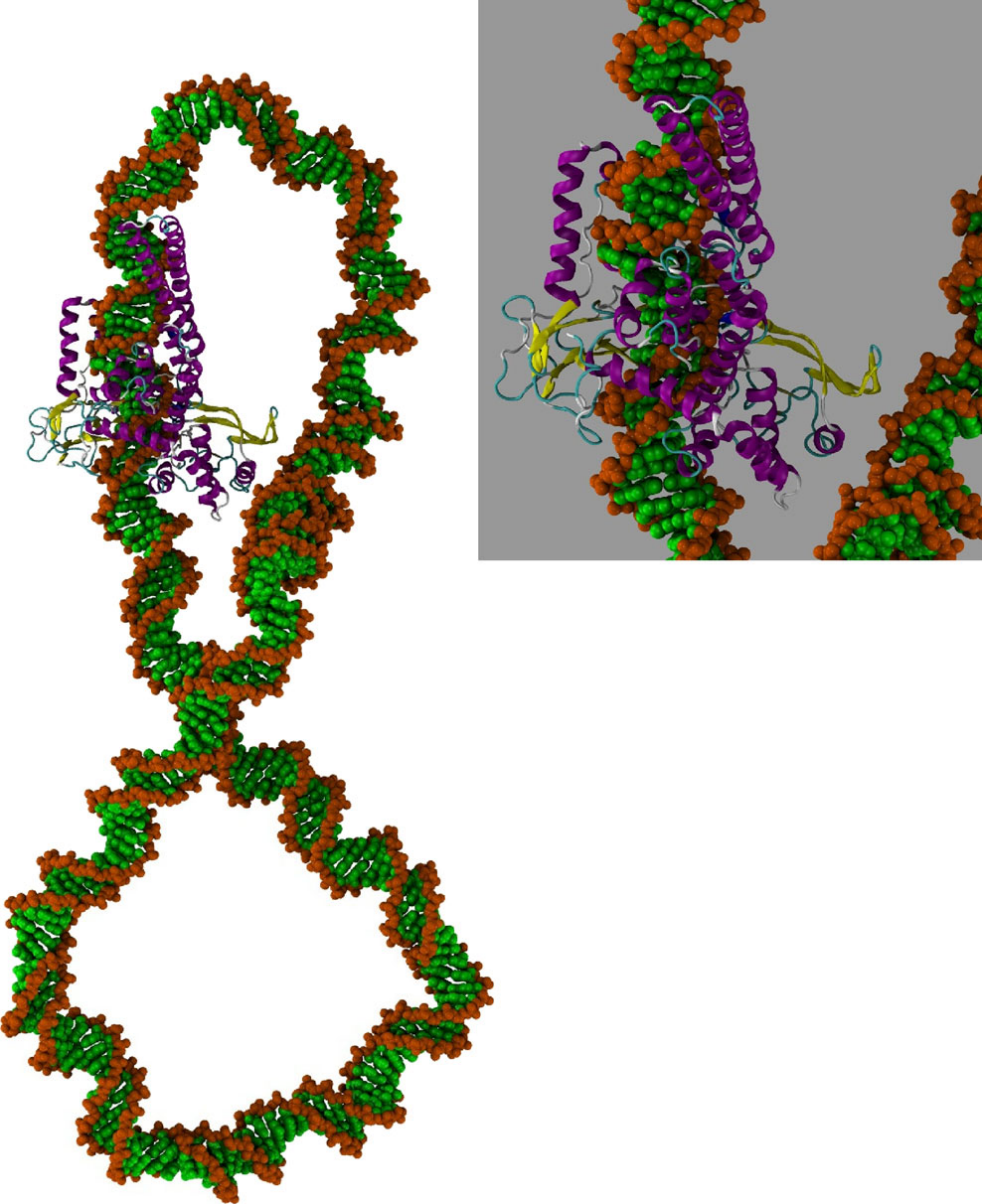
The human topoisomerase 1B complex with supercoiled DNA showing an additional non-specific protein–DNA interaction within the plectoneme (D’Annessa et al. 2014)

DNA architectural proteins are characterised by their net positive charge. Therefore, from a physical standpoint they are expected to behave as “complex cations”, as in much the same way as cationic counterions, but with a significantly larger size and distinctive sequence preferences. Similar to simple salt cations, complex DNA topologies have been shown to promote additional protein–DNA interactions that would be largely absent in linear DNA sequences. MD simulations of human topoisomerase IB bound to a plectonemic DNA minicircle, as shown in Fig. 3, detected an additional DNA binding site which results in a bridge between positively charged lysine residues on the protein surface far from the canonical DNA binding site and a distant non-specific site on the opposite site of the minicircle (D’Annessa et al. 2014). Within the “complex counterion” view of protein–DNA interactions, the formation of protein bridges between distal sites but on adjacent strands of plectonemic DNA might be expected for any protein that possesses positively charged residues on its surface, so long as they are in a favourable orientation relative to the DNA binding site (e.g. on the opposite side). An additional similarity between simple counterions and the “complex counterionic” structural DNA binding proteins is their ability to locally increase the flexibility of the DNA and influence the partitioning between twist and writhe by inducing tight bends, such as by the bacterial nuclear architectural proteins HU or FIS (Travers and Muskhelishvili 2007). However, DNA-binding proteins have a far greater structural and chemical diversity compared to counterions of simple salts. This can be associated with sequence-specific and co-operative interactions between the various DNA-binding species which can regulate gene expression, as we now describe.

### Coupling of protein binding to topology and transcription control

Unlike conventional transcription factors, which bind to specific sequences, bacterial architectural proteins such as FIS, IHF, H-NS and HU impose transcriptional control by their influence on the global shape of supercoiled DNA. The factor of inversion stimulation (FIS) is a family of architectural proteins found in prokaryotes that organises the bacterial chromosome and stabilises plectoneme branching (Schneider et al. 2001). FIS binds at hundreds of sites in *E. coli* DNA with weak specificity through poorly conserved sequence motifs characterised by high A/T content (Cho et al. 2008). An example of its control of transcription is provided by the *tyrT* promoter in *E. coli.* Around three FIS dimers bind to the promotor region and stabilise a DNA microloop, which then facilitates transcription initiation (Muskhelishvili et al. 1997). Another example of the coupling between nuclear binding proteins, DNA topology and transcription involves the architectural protein HU (heat-unstable nucleoid protein). Although HU binds to DNA in a largely sequence-independent manner, it binds with much higher affinity to distorted DNA molecules, such as nicked, bent, gapped or AT-rich DNA (Kamashev and Rouviere-Yaniv 2000). HU can bind to pairs of juxtaposed DNA segments so that a writhed plectonemic conformation is favoured, which enables transcription to be initiated from the bent apical ends where the DNA is prone to local melting (Travers and Muskhelishvili 2007). These examples show how topology-dependent binding enables otherwise non-specific DNA-binding proteins to discriminate between different sites in writhed DNA.

A rich array of complex higher-order structures have been observed by AFM for plasmids in the presence of mixtures of these structural proteins (Maurer et al. 2009). X-ray crystallography has also shown that the HU/DNA protein complex can exist as a left-handed spiral filament in which the grooves act as a DNA-binding pocket and the DNA is negatively supercoiled (Guo and Adhya 2007). This degree of potential structural organisation is reminiscent of the situation in eukaryotes; however, the structure of the nucleosome and its higher-order organisation is additionally determined by a complex interplay of epigenetic factors and the occurrence of cell signals that switch on the chromatin-remodelling machinery (Magueron and Reinberg 2011).

### Protein binding to supercoiled DNA loops

In prokaryotes, non-specific architectural proteins have been shown to couple the global DNA topology to sequence-selective recognition in transcription regulation by controlling DNA looping. DNA looping is a ubiquitous regulatory architecture affecting such fundamental processes as transcription, replication and recombination that require communication between distal chromosomal sites. A series of experiments and simulations have confirmed that architectural proteins, which discriminate between different sites by recognising specific DNA topologies rather than sequences (such as HU), bind pre-formed DNA loops with higher affinity, which correspondingly increases the looping probability (Becker and Maher 2015; Lia et al. 2003; Swigon et al. 2006; Wei et al. 2014). Single-molecule manipulation and tethered particle tracking experiments have shown that the ability of the *lac* (Normanno et al. 2008; Ding et al. 2014) and λ (Norregaard et al. 2013) repressor proteins to mediate DNA looping is promoted by supercoiling because compacting the DNA increases the likelihood that the distant binding sites will come sufficiently close together. In the case of the λ repressor, the transition between looped and unlooped states was found to be sharper for supercoiled DNA relative to linear DNA templates, implying that supercoiling amplifies the biochemical signal from protein binding in this instance (Norregaard et al. 2013).

The dependence of DNA looping on distant site juxtaposition probabilities implies that the global dynamics of a closed DNA topology is important. Cryo-electron microscopy studies and MD simulations of 336-bp DNA minicircles have shown that supercoiled DNA loops are surprisingly structurally diverse, with a broad range of conformers—from highly compacted structures to open circles—observed even for a single topoisomer (Irobalieva et al. 2015). While any sequence dependence in global topology that arises from the intrinsic mechanics of the DNA molecule, for example the preference of a highly flexible TA step to be present at the bent apices relative to a stiff GG motif, will indeed favour a unique global configuration, thermal fluctuations continuously drive the DNA molecule to explore different conformations, a process that will change which segment of the sequence lies at the apical loops and continuously bring different distant sites into close proximity. Therefore, any change in the flexibility of the DNA might be expected to affect protein–protein and protein–DNA interactions that involve bridging between distant sites. The differences between looping probabilities and cyclisation or nucleosome formation have been highlighted by single molecule-tethered particle motion assays, which found that poly(dA:dT)-rich DNA has a particularly high looping probability in spite of its low nucleosome affinity (Johnson et al. 2013). This observation suggests an exquisite sensitivity to the detailed “boundary conditions” associated with the topological restraint of the DNA within the complex, which may originate in part from the dependence of contact probabilities on DNA conformational flexibility.

### Importance of topology to long-range DNA looping

DNA looping over far longer sections of DNA can be required to bring enhancer sequences, which contain multiple transcription factor binding sites, into contact with their cognate promotors during transcription activation. Although enhancers may be situated long distances apart in sequence (i.e. >10 kb) from a promotor, the 3D organisation of the chromosome can place them close together in space to form “active chromosome hubs” which are poised to act co-operatively given an appropriate biochemical signal (Ong and Corces 2011). The driving force for compaction of the DNA into separate globular domains has not been determined conclusively; however, computer models that treat the DNA as a simple flexible polymer have indicated that multiple protein–DNA interactions (Barbieri et al. 2012) and supercoiling (Benedetti et al. 2014; Le et al. 2013) are both likely to play a role. Intriguingly, coarse-grained simulations have shown that the clustering of binding proteins on DNA can be entropically driven. Cluster formation minimises the loss of entropy caused by confining the DNA into loops (Brackley et al. 2013), which indicates that the thermodynamics of supercoiled DNA and its protein complexes can play a role in 3D genome organisation in unexpected ways.

The top level of the hierarchical organisation of the nuclear material is finally determined by the interactions between the DNA and those structural elements that fix the genome to a given point in 3D space. Studies which monitored changes in the nuclear domain architecture in response to depleted levels of the structural proteins cohesin and CTCF (11-zinc finger protein) found that cohesin was necessary for maintaining the compact structure of individual topological domains, while CTCF was necessary to maintain the boundaries between these domains (Zuin et al. 2014). The location of these fixed boundaries is also likely to control the distribution of supercoiling throughout the genome, enabling different levels of supercoiling to be maintained in different regions of the DNA at any given time. DNA loops that have been locked into place through the formation of lac, gal and lambda repressor protein complexes between distant sites can be sufficiently robust to divide the molecule into two independent topological domains, thereby creating a barrier that prevents supercoiling diffusion through the plasmid (Ding et al. 2014; Leng et al. 2011); this barrier has been shown to play a regulatory role in expression of the *lac* operon in *E. coli* (Fulcrand et al. 2016). The formation of locally melted DNA due to high levels of negative superhelical stress are also likely to act as barriers to supercoil diffusion because of their reduced ability to transmit superhelical stress. Through supercoiling, the global arrangement of the genome is communicated across multiple levels of hierarchical organisation back to the DNA structure itself.

## Untangling the importance of supercoiling in gene regulation

We have discussed the concept of supercoiling-mediated coupling between the multiple length-scales associated with hierarchical nuclear structural organisation. We have shown how supercoiling acts as an amplification device for small local changes in structure or flexibility, as these can be sufficient to affect the global structure and dynamics of far larger sections of topologically closed DNA. Conversely, supercoiling is controlled by the location of attachment points which fix the nuclear material in 3D space and can thereby communicate messages at a cellular level (for example during cell cycle regulation) down to the level of an individual gene promotor.

Quantifying the distribution of supercoiling throughout the genome and the detection of the transient torsional stresses that are transmitted through DNA by DNA processing proteins, such as the transcription complex, is extremely experimentally challenging. Supercoiling is consequently not as well established as a controller of transcription as conventional biochemical interactions, such as activa-tion or repression by sequence-selective DNA binding proteins. However, it is apparent that there is sufficient complexity and potential for information transfer through DNA mechanics for supercoiling to act as a valuable contributor to global gene regulation, acting in conjunction with biochemical signals from transcrip-tion factors and other external regulatory proteins.
